# Initial experience with sacral neuromodulation for the treatment of lower urinary tract dysfunction in Brazil

**DOI:** 10.1590/S1677-5538.IBJU.2014.0603

**Published:** 2016

**Authors:** Luis Augusto Seabra Rios, Marcio Augusto Averbeck, Wagner França, Carlos Alberto Ricetto Sacomani, Fernando G. Almeida, Cristiano Mendes Gomes

**Affiliations:** 1 Deparamento de Urologia, Hospital Albert Einstein, SP, Brasil; 2 Departamento de Urologia, Hospital Moinhos de Vento, Porto Alegre, RS, Brasil; 3 Universidade Federal de São Paulo, Escola Paulista de Medicina, SP, Brasil; 4 Departamento de Urologia, AC Camargo Hospital, SP, Brasil; 5 Departamento de Urologia, Faculdade de Medicina da Universidade de São Paulo, SP, Brasil

**Keywords:** Sacrum, Urinary Tract, Therapeutics

## Abstract

**Objectives:**

We report on the short-term outcomes of sacral neuromodulation (SNM) for treatment of idiopathic lower urinary tract dysfunction in Brazil (procedures performed before 2014).

**Materials and Methods:**

Clinical data and surgical outcomes of patients who underwent SNM staged procedures were retrospective evaluated. Urological assessment included a focused medical history and physical examination, measurement of postvoid residual volumes, urodynamics, and bladder diaries. A successful test phase has been defined by improvement of at least 50% of the symptoms, based on bladder diaries.

**Results:**

From January 2011 to December 2013, eighteen consecutive patients underwent test phase for SNM due to refractory overactive bladder (15 patients), non-obstructive chronic urinary retention (2 patients), and bladder pain syndrome/interstitial cystitis (1 patient). All patients underwent staged procedures at four outpatient surgical centers. Mean age was 48.3±21.2 (range 10-84 years). There were 16 women and 2 men. Median follow-up was 3 months. Fifteen patients (83.3%) had a successful test phase and underwent implantation of the pulse generator (IPG). Median duration of the test phase was 7 days (range 5–24 days). Mean age was 45.6±18.19 years in responders versus 61.66±34.44 years in non-responders (p=0.242). Mean operative time (test phase) was 99±33.12 min in responders versus 95±35 min for non-responders (p=0.852). No severe complications were reported.

**Conclusion:**

SNM is a minimally invasive treatment option for patients with refractory idiopathic lower urinary tract dysfunction. Our initial experience with staged technique showed that tined-lead electrodes yielded a high rate of responders and favorable clinical results in the short-term follow-up.

## INTRODUCTION

Sacral neuromodulation (SNM) is an established alternative for the treatment of idiopathic lower urinary tract dysfunction in patients who failed previous therapies. Although its exact mechanism of action is not fully understood, it is believed that the therapeutic benefits arise from the effects of electrical stimulation on afferent and efferent nerve fibers connecting the pelvic viscera and the spinal interneurons to the central nerve system.

SNM has been studied as a third-line treatment for idiopathic lower urinary and bowel tract dysfunctions, including overactive bladder syndrome, non-obstructive chronic urinary retention, anal incontinence, and chronic pelvic pain.

In urology, the most common indication for this therapy is the overactive bladder syndrome (OAB), which is defined by the International Continence Society (ICS) as urgency, with or without incontinence, usually accompanied by uri-nary frequency and nocturia, in the absence of urinary tract infection or other pathological metabolic conditions (1).

OAB has a negative impact on the quality of life and affects individuals of both genders, in different age groups (2). It is estimated that the prevalence of OAB symptoms in Brazil is 18.9% (3). Patients tend to social isolation and are at increased risk for developing depressive symptoms. Nocturia, which is present in most patients, may be associated with impaired sleep quality and increased risk of falls and fractures, particularly in the elderly (4). Multiple interventions have been described for the treatment of patients with OAB symptoms (5). Conservative treatment is the first line of treatment and includes behavioral treatment, bladder training, and pelvic floor muscle exercises (PFME). Antimuscarinics and beta-3–agonists are regarded as second-line treatments (2, 5, 6). Despite the documented efficacy of these treatment options, many patients do not significantly improve upon conservative therapies. Refractory OAB presents as a complex clinical condition in urological practice. Current third–line treatment options for refractory OAB include injection of botulinum toxin in the detrusor, percutaneous tibial nerve stimulation (PTNE), and sacral neuromodulation (SNM).

Another approved urological indication for SNM is chronic non-obstructive urinary retention. Urinary retention without an identifiable urological cause presents a therapeutic challenge. Patients with non-obstructive chronic urinary retention usually have to rely on intermittent self-catheterization, which significantly affects quality of life and may be associated with complications, such as urinary tract infections and urethral trauma. Even though its physiopathology is not well understood, this disorder seems to be related to a primary failure of relaxation of the striated urethral sphincter (7). Not only a typical clinical history, but also the evaluation of the sphincter volume and electromyography may be determining factors for the diagnosis. Sacral neuromodulation may improve many of these patients (7).

Although SNM has gained increased worldwide acceptance for the treatment of patients with refractory OAB and non-obstructive urinary retention, in Brazil this therapy has had a modest and hesitant experience. Brazilian regulatory authorities have approved it as of August/2004. However, it was only after January/2014 that its coverage by private health insurance companies became mandatory. In addition, the technical and institutional support by the manufacturer (Medtronic Minneapolis, MN, USA) significantly improved since then. As a result, increasing interest in the therapy has been observed. Before 2014, only a few Interstim^TM^ implants for different indications had been made in Brazil. We herein report on our initial experience with SNM for the treatment of lower urinary tract dysfunction (procedures performed before 2014).

## MATERIALS AND METHODS

This is a retrospective study to evaluate the short-term outcomes of SNM for the treatment of idiopathic lower urinary dysfunction in Brazil. Clinical data and surgical outcomes of patients from 4 medical centers were analyzed.

OAB was defined according to the ICS criteria (1).

Chronic non-obstructive urinary retention was defined as chronic urinary retention and secondary need for intermittent catheterization in the absence of anatomical cause of obstruction. Fowler's syndrome is the main condition that leads to chronic non-obstructive urinary retention and it is caused by the failure to relax of the striated urethral sphincter during voiding in young women [27]. Urodynamics with surface perineal electromyography and, when available, videourodynamics have been used to confirm this diagnosis. Exclusion criteria were evidence of urethral stenosis, pelvic organ prolapse or neurological diseases.

Urological assessment at baseline included a focused medical history and physical examination, urinary tract ultrasound with measurement of postvoid residual volumes, multichannel uro-dynamics, and a three-day bladder diary. Bladder diaries included number of micturitions, urine volume, episodes of urgency and urinary incontinence over 24h. Catheterized urine volumes and postvoid residual volumes were also recorded in patients with chronic non-obstructive urinary retention, who were on clean intermittent catheterization. Visual analogic scale (1 to 10 points) has been used to subjectively evaluate pain in patients with interstitial cystitis.

Data were expressed as mean (or medians) ± standard deviation (minimum and maximum values). Mann-Whitney test was used to statistically compare continuous variables and chi-square test to compare categorical variables. IBM SPSS^®^ Statistics version 22 for Mac (SPSS Inc., Chicago, IL, USA) was used for statistical analysis. All differences with a p value less than 0.05 were considered statistically significant.

### Surgical Technique – Staged Implantation

All patients underwent SNM staged implantation technique. This consists of a two–phase procedure, including a test phase during which the improvement of symptoms is evaluated, allowing deciding whether this therapy is effective or not. Those patients with a successful test phase proceeded with the second stage, which is the implantation of the programmable pulse generator (IPG). A successful test phase was considered when an improvement of at least 50% of the symptoms based on the bladder diaries was obtained and the patient was satisfied with this improvement. The test phase typically takes one to two weeks and should be as short as possible to minimize the risk of infection. The implant of the quadripolar electrode (tined-lead electrode) followed the well-established technique that has been previously published (8). Therapeutic failure has been defined as symptomatic improvement <50%, which was an indication for electrode removal.

All interventions were performed with the patients lying on the prone position and under cardiovascular monitoring, guided by posteroanterior and lateral fluoroscopy. The procedures were performed with local anesthesia with 20-40mL of 1% lidocaine and mild conscious intravenous sedation. Patients received either intravenous cefazolin (1g) or ciprofloxacin (400mg) just before the procedure, and oral ciprofloxacin (500mg PO twice a day) for five days.

Patients were discharged home three hours after the procedure with instructions on how to take care of the electrode and the external pulse generator. During the test phase, patients were re-evaluated at the office every 2-4 days, and adjustments in the electrical stimulation program were performed as appropriate.

If the patient was a ‘responder’, the definitive pulse generator (IPG) was implanted subcutaneously in the supero-lateral quadrant of the buttock (pocket site), through the previously made incision. Patients with a failed test phase underwent electrode removal under local anesthesia.

## RESULTS

From January 2011 to December 2013, eighteen consecutive patients underwent SNM test phase due to refractory overactive bladder (15 patients; 12 patients with ‘wet’ OAB and 3 patients with ‘dry’ OAB), non-obstructive chronic urinary retention (2 patients), and bladder pain syndrome/interstitial cystitis (BPS/IC) (1 patient). All patients underwent staged procedures at four outpatient surgical centers (none required hospitalization). Mean age was 48.3±21.2 (range 10-84 years). There were 16 women and 2 men. Baseline characteristics are described in Table-1. Median follow-up was 3 months (range 1 to 5 months).

**Table 1:** Baseline characteristics.

Initials	Age*	Gender	Comorbidities	Medication	Previous urological treatments	Urological Diagnosis
D.M.	71	Female	Hypertension, nocturnal polyuria	Enalapril	Oxybutynin, solifenacin, desmopressin, tibial nerve stimulation	OAB¥ without urinary incontinence, nocturnal polyuria
M.G.	37	Female	Juvenile Rheumatoid Arthritis	Methotrexate	Oxybutynin, solifenacin, tolterodine, darifenacin	OAB with urinary incontinence
E.M.G.	18	Female	None	None	Oxybutynin, solifenacin	OAB with urinary incontinence
M.S.B.	62	Female	Hypertension	Enalapril	Oxybutynin, solifenacin	OAB with urinary incontinence
M.B.O.	32	Female	None	None	Oxybutynin, solifenacin, tibial nerve stimulation, onabotulinum toxin	OAB with urinary incontinence
A.V.	48	Male	None	None	Oxybutynin, tolterodine, tibial nerve stimulation, bladder augmentation, intermittent catheterization	OAB without urinary incontinence
J.D.	79	Male	Previous coronary artery disease	None	TURP**, tolterodine, onabotulinum toxin	OAB with urinary incontinence
M.L.G.	56	Female	Previous non-muscle-invasive bladder cancer	None	Oxybutynin, tolterodine	OAB with urinary incontinence
D.A.A.	59	Female	None	None	Tolterodine, solifenacin, imipramine	OAB with urinary incontinence
M.A.G.	59	Female	Hypertension, Diabetes Mellitus, Hypothyroidism	Enalapril, Metformin, Levothyroxine	Oxybutynin, tolterodine, onabotulinum toxin	OAB with urinary incontinence
M.P.A.F.	64	Female	Dyslipidemia	None	Darifenacin, oxybutynin	OAB with urinary incontinence
A.M.C.l.	53	Female	Thalassemia	None	Tolterodine, solifenacin, onabotulinum toxin	OAB with urinary incontinence
T.P.S.S.	35	Female	Endometriosis	Morphine	Intermittent catheterization	BPS/IC§
D.P.S.	26	Female	Thymoma, hyperhidrosis	None	Intermittent catheterization	Chronic urinary retention
M.T.M.	54	Female	None	None	Oxybutynin, solifenacin, onabotulinum toxin	OAB without urinary incontinence
M.M.G.	10	Female	Neonatal intracranial hemorrhage, ventriculo-peritoneal shunt (at age 2). Cerebral palsy.	None	Surgery for vesico-ureteral reflux, oxybutynin, prophylactic antibiotic, physiotherapy and transcutaneous electrical stimulation	OAB with urinary incontinence
M.C.R.	84	Female	Chronic renal failure	None	Intermittent catheterization/prophylactic antibiotic	Chronic urinary retention, nocturnal polyuria
B.M.	22	Female	None	None	Oxybutynin, tolterodine, darifenacin, solifenacin, physiotherapy, botulinum toxin	OAB with urinary incontinence

* Years

** **TURP =** Transurethral resection of the prostate

¥ **OAB =** overactive bladder syndrome

§ **BPS/IC =** bladder pain syndrome/interstitial cystitis

Among patients with OAB, urodynamic data showed increased bladder sensation (mean volume at first desire=76.25±59.89mL; 40-250mL). All but one patient had demonstration of detrusor overactivity during cystometry (n=14). Mean bladder capacity was 287.85±106.42mL (150-500mL). Available preoperative urodynamic data of OAB patients are presented in Table-2.

**Table 2:** Available preoperative urodynamics data.

Initials	Post-void residual (mL)	First Desire (mL)	Detrusor Overactivity	Cystometric Capacity (mL)
D.M.	5	40	None	200
M.G.	10	50	Yes	250
E.M.G.	0	50	Yes	180
M.S.B.	10	100	Yes	300
M.B.O.	0	60	Yes	220
A.V.	40	80	Yes	420
J.D.	50	40	Yes	400
D.A.A.	15	***	Yes	300
M.A.G.	5	60	Yes	150
M.P.A.F.	60	40	Yes	380
A.M.C.I.	19	250	Yes	500
M.T.M.	10	***	Yes	230
M.M.G.	30	110	Yes	170
B.M.	5	35	Yes	330

Available data of patients diagnosed with refractory overactive bladder (there was 1 unavailable urodynamic examination on chart review)

*** Missing data

Fifteen patients (83.3%) had a successful test phase and underwent implantation of the IPG. Among the three patients who failed the test phase, two had OAB and one had urinary retention. Duration of test phase varied according to the patient's response to treatment. Those who did not have a good response in the first days after the implant required an extended period of evaluation, during which different programming settings were tested. Median duration of the test phase was 7 days (range 5–24 days). Mean operative time during test phase was 98.33±32.4 min (40–130 min). Outcomes of the test phase are shown in Table-3.

**Table 3:** Outcomes of the test phase according to urological condition.

Urological condition	n (%)	Mean procedure time (min)	Success (%)
Refractory OAB¥	15 (83.3)	99.3	13 (86.7)
Urinary retention	2 (11.1)	82.5	1 (50.0)
BPS/IC§	1 (5.6)	120.0	1 (100)
Total	18 (100)	98.3	15 (83.3)

¥ **OAB =** overactive bladder syndrome

§ **BPS/IC =** bladder pain syndrome/interstitial cystitis

After IPG implant all the patients continued having good clinical responses at short–term follow-up. Re-programming was done in most patients as a mean of finding the best clinical scenario with the least energy spending.

Both patients with urinary retention in our series had failed a trial of alpha-blocker treatment and were not on medications during baseline evaluation. One out of two patients diagnosed with idiopathic chronic urinary retention and the only patient with BPS/IC had successful test phases. The responsive patient with chronic urinary retention had an 80% reduction of post-void residual postoperatively. This patient did not need intermittent catheterization in the short-term follow-up. In the other patient with chronic urinary retention, S3 stimulation was not successful and the tined-lead was removed 10 days after implantation. The patient with BPS/IC had a 60% improvement in the storage urinary symptoms and reported 90% of improvement in chronic pain during the test phase. Due to this positive response, IPG was implanted 14 days after the initial procedure.

Mean age was 45.6±18.19 years in responders versus 61.66±34.44 years in non-responders (p=0.242). Mean operative time (test phase) was 99±33.12 min in responders versus 95±35 min for non-responders (p=0.852).

There were no Clavien 2 or higher complications. When present, pain was mild and no patient needed narcotic analgesics. No infectious complications were observed.

## DISCUSSION

SNM has been approved by the Food and Drug Administration (FDA) for the treatment of refractory urinary dysfunction since the late 1990s and over 150.000 implants have been made throughout the World. In Brazil, this therapy was first approved by regulatory authorities in August/2004, but only a few implants had been made up to 2014. As of January/2014, the coverage of SNM by private health insurance companies became mandatory and it is expected that SNM should progressively attract patients and urologists alike. For this reason, we found it would be appropriate to report on our initial experience with this exciting therapy.

Our series is consistent with most retrospective studies reporting on a global SNM experience, which included a predominant population of refractory OAB patients and fewer patients with urinary retention or BPS/IC (9, 10).

We used staged procedures for all patients. In Brazil, the electrodes for percutaneous nerve evaluation (PNE) are not available, which makes mandatory the selection of the staged procedure with the implantation of a tined-lead electrode initially. The staged procedure technique has been described as a successful strategy to improve the chance of response during the stimulation test (first stage). Borawski et al. randomized 30 patients to electrical stimulation test with different electrodes and showed that the chance of identifying responders is higher with the tined-lead electrode compared to the conventional percutaneous nerve evaluation (PNE) electrode (88% versus 46%, P=0.02) (11).

The high rate of responders presented in our study is also consistent with the medical literature. An European multicenter study evaluated 94 patients with different types of dysfunctional voiding and identified 72 patients responsive to electrical stimulation test with tined-leads (76.6%) (12). More recently, Chartier-Kastler et al. published a prospective study conducted between 2003 and 2009 in 44 French centers, including 1418 patients who underwent implants for overactive bladder (1170 patients), idiopathic urinary retention (151 patients), and other disorders (97 patients). In OAB patients, clinical improvement higher than 50% was described in 84.8% of cases (13).

The duration of the test phase is an important aspect of the treatment. Kessler et al. prospectively evaluated 20 patients undergoing prolonged test phase for a minimum of 14 days and identified 80% of responders. These patients received suppressive antibiotics and had no major complications (14).

Some of our patients were kept in the test phase for more than one week. This strategy allowed us to test different electrical stimulation settings with satisfactory outcomes. However, it was not possible to establish any association between the duration of the test phase and the success/failure (responders versus non-responders).

Due to the small population sample in our study, we were not able to evaluate predictors of response. Factors such as age and operative time were not predictors of clinical response to SNM in our series (p>0.05). Several studies have tried to identify clinical factors to predict which patients are most likely to benefit from SNM (8, 15–20). Amundsen et al. investigated 105 patients with urgency urinary incontinence that underwent electrical stimulation with PNE test, of which 55 (52%) received the IPG implant. Age above 55 years was associated with a greater chance of success (65% versus 37%; p<0.05). Regardless of age, the presence of 3 or more comorbidities and the diagnosis of a neurological disease were predictors of failure (18). There is also evidence that emotional distress and psychiatric illness are associated with a reduced chance of response to the stimulation test and a higher incidence of reoperations after IPG implant (19, 20).

Urodynamics does not seem to be helpful in selecting the best candidates for the SNM. There is some evidence to suggest that urodynamic evaluation cannot predict the response to SNM test phase or estimate the chance of success after the IPG implant. A non-randomized study found that detrusor overactivity during cystometry does not correlate with the possibility of response during test phase (16). In our series, urodynamics were obtained in all patients at the baseline evaluation as part of a comprehensive urological workup. However, we were not able to evaluate its use as a prognostic factor due to the small population of our study. Most of our OAB patients had demonstration of detrusor overactivity during cystometry.

Our study included only a short-term evaluation period (follow-up). According to data from various long-term prospective studies, clinical improvement with SNM is maintained after up to 5 years in most patients. Success rates of 60-77% have been reported (7, 15, 21–27).

Previously published systematic reviews demonstrated SNM broad clinical utility. Brazelli et al. systematically reviewed studies published between 1996 and 2003, and found success rates between 67 and 80% for OAB patients, with sustained results after a follow-up of 3-5 years (17). Kessler et al. reviewed the outcomes of 30 studies, including 357 patients. Success rates were 68% (95% CI: 50-85%) for the test phase and 92% (95% CI: 81-98%) after IPG implantation. This systematic review did not exclude 88 patients with neurogenic conditions in the analysis (28).

In our series only one patient with a neurological condition was included (cerebral palsy), which has been regarded as a good responder during the test phase and underwent IPG implantation. International experience with neurological patients is limited to small case series (28).

No significant complications were observed in our series. Hijaz et al. reviewed the complications of SNM in 214 patients who had staged procedures (29). One hundred and sixty-one patients (75.5%) were regarded as responders during the test phase and underwent IPG implantation. In seventeen patients (10.5%) the device had to be completely removed due to infection (n=8) or absence of clinical response (n=9). Twenty-six patients (16.1%) underwent surgical revision due to attenuation of the response (n=17), infection (n=4), pain at the IPG site (n=4), and electrode migration (n=1). Other studies also showed that surgical revision rates accumulate over time (30, 31).

There are major limitations in the present study, including the small number of participants and the fact that our series of 18 patients was shared among four medical centers, which did not enable any of the implanters to advance significantly in the learning curve before 2014. In addition, the follow-up of the patients was short and did not follow a structured prospective protocol. Moreover, we included patients with different urological conditions. Despite of these inherent limitations, our results are consistent with those of previously published studies and represent the initial experience of SNM in Brazil (14–17). Our series included patients with refractory OAB (n=15), urinary retention (n=2) and, interstitial cystitis (n=1). In such a heterogeneous population, no validated questionnaires could be used to properly assess symptoms. Lower urinary tract symptoms have been evaluated by means of focused clinical history (based on their clinical condition) and bladder diaries (baseline and test phase).

## CONCLUSIONS

SNM is a minimally invasive treatment option for patients with refractory idiopathic lower urinary tract dysfunction. Our initial experience with staged technique showed that tined-lead electrodes yielded a high rate of responders and favorable clinical results in the short-term follow-up.
